# Supplementary material for: PyFuRNAce: an integrated design engine for RNA origami
Source: Nat Commun. 2025 Dec 1;16:10815. doi: 10.1038/s41467-025-66290-x (PMC12669765; doi:10.1038/s41467-025-66290-x)
Supplement: Supplementary file 1 — Supplementary Information [file 41467_2025_66290_MOESM1_ESM.pdf]

Supplementary Information
PyFuRNAce: An Integrated Design Engine for RNA Origami

Luca Monari[1,2], Ina Braun[2,3,4], William Verstraeten[1,2], Erik Poppleton[1,2,5], and Kerstin Göpfrich[1,2,*]

[1]Center for Molecular Biology of Heidelberg University (ZMBH), Berliner Str. 45, 69120 Heidelberg, Germany
[2]Max Planck Institute for Medical Research, Jahnstraße 29, 69120 Heidelberg, Germany
[3]Max Planck School Matter to Life, Jahnstraße 29, 69120 Heidelberg, Germany
[4]Max Planck Institute for Dynamics and Self-Organization, Am Faßberg 17, 37077 Göttingen, Germany
[5]Max Planck Institute for Polymer Research Ackermannweg 10, 55128 Mainz, Germany
[*]Corresponding author: Kerstin Göpfrich, email: k.goepfrich@zmbh.uni-heidelberg.de

Contents

1 Supplementary Figures **2**
1.1 Figure S1: Diagram of the dependencies of the Design page and Motif classes 2
1.2 Figure S2: Diagram of the dependencies of the Generate / Prepare pages and the App subpackage 3
1.3 Figure S3: Screenshots of the Generate page 4
1.4 Figure S4: Screenshot of the Convert page 5
1.5 Figure S5: Screenshot of the Prepare page 5
1.6 Figure S6: Agarose gel electrophoresis and plate reader measurements 6
1.7 Figure S7: AFM of filament-forming RNA origami 7
1.8 Figure S8: AFM of large rectangular RNA origami 8
1.9 Figure S9: Yield estimation 9
1.10 Figure S10: RNA droplet variants 10

2 Supplementary Videos **11**
2.1 Video S1: Tutorial video 11

3 Supplementary Notes **11**
3.1 Note S1: Comparison of RNA design tools 11
3.2 Note S2: Automated primer design 12
3.3 Note S3: Software limitations and workarounds 13
3.4 Note S4: OxRNA Molecular Dynamics Simulation Setup 15
3.5 Note S5: RNA nanostructure scripts 16

1 Supplementary Figures

1.1 Figure S1: Diagram of the dependencies of the Design page and Motif classes

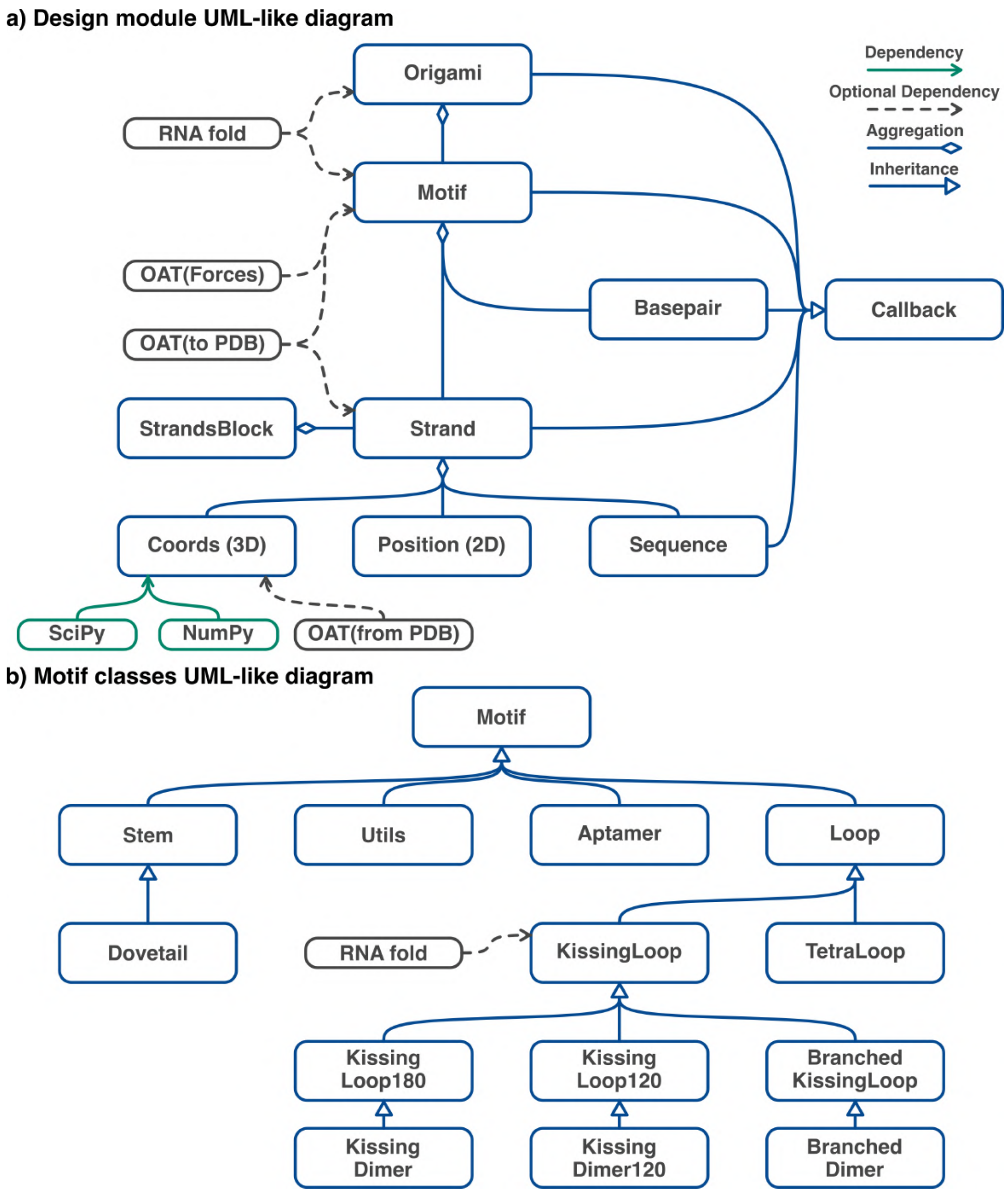

Figure S1: **a)** UML-like diagram representing the class structure of the Design sub-package of pyFuRNAce. The dependencies are shown in smaller boxes, with green indicating required dependencies and grey indicating optional dependencies. **b)** UML-like diagram representing the currently available Motif classes in pyFuRNAce. The RNAfold dependency is needed only when using a custom sequence in a Kissing Loop to calculate the respective energy.

1.2 Figure S2: Diagram of the dependencies of the Generate / Prepare pages and the App subpackage

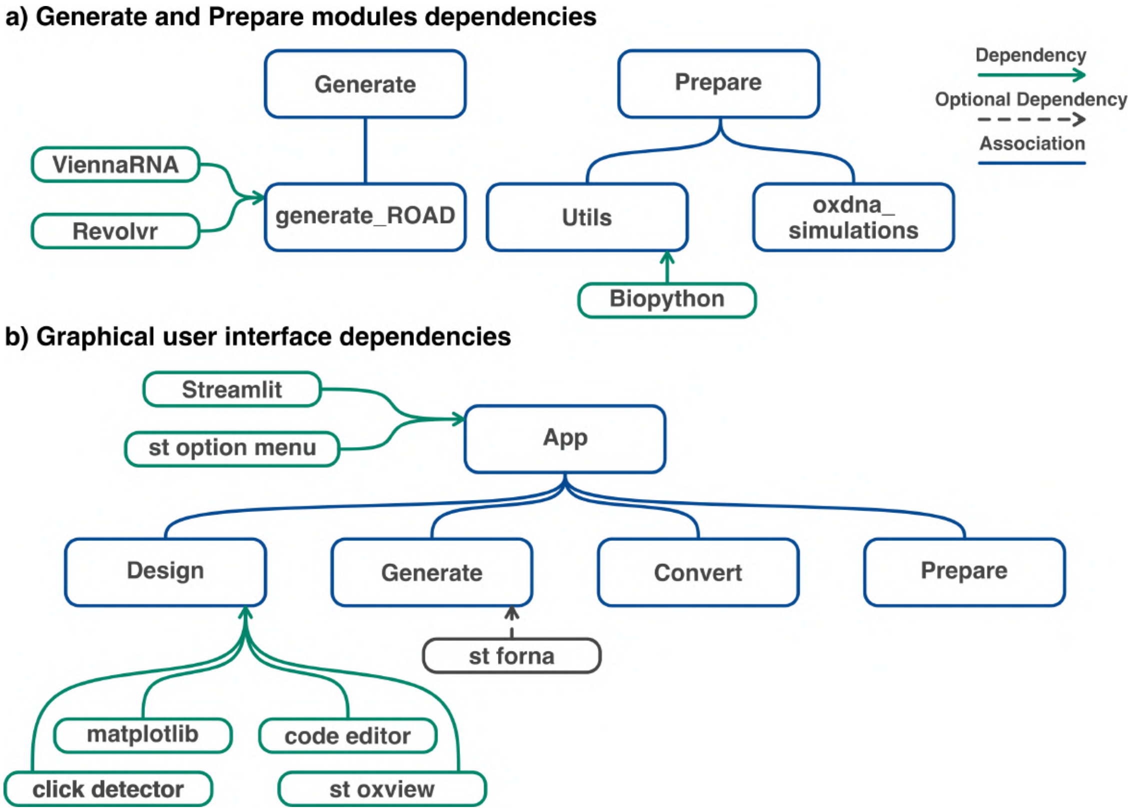

Figure S2: **a)** General diagram to represent the organisation and dependencies of the Generate and Prepare subpackages of pyFuRNAce. **b)** General diagram to represent the organisation and dependencies of the pyFuRNAce graphical user interface (GUI), in the App subpackage. The GUI Design page depends on the pyFuRNAce Design subpackage; the GUI Generate page depends on the pyFuRNAce Generate subpackage, while the GUI Convert and Prepare pages depend on the pyFuRNAce Prepare subpackage.

1.3 Figure S3: Screenshots of the Generate page

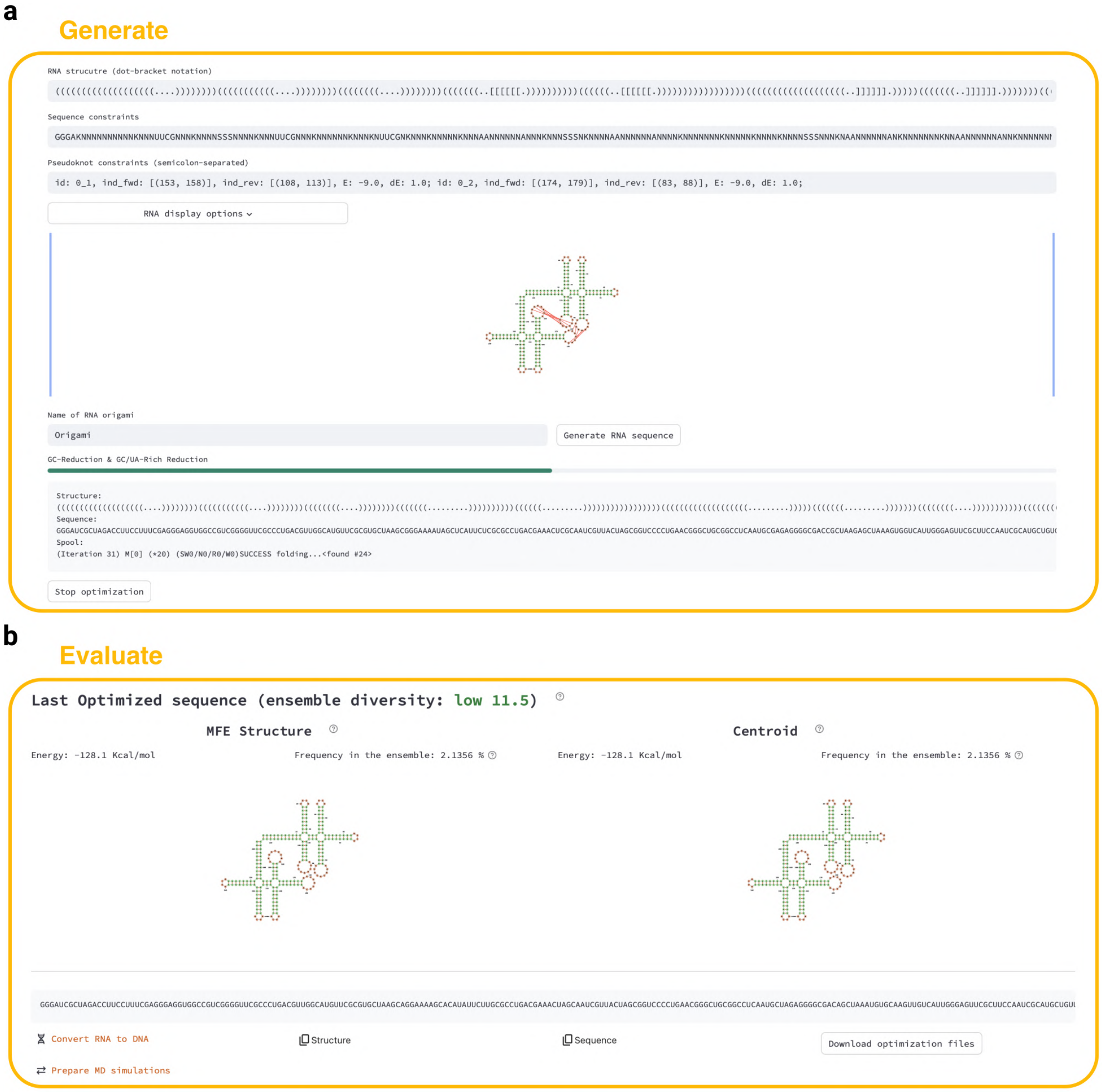

Figure S3: **a)** Screenshot of the pyFuRNAce 'Generate' page. **a)** Optimization of an RNA origami sequence. **b)** The final thermodynamic parameters calculated with ViennaRNA to evaulate the folding of the structure [1, 2].

1.4 Figure S4: Screenshot of the Convert page

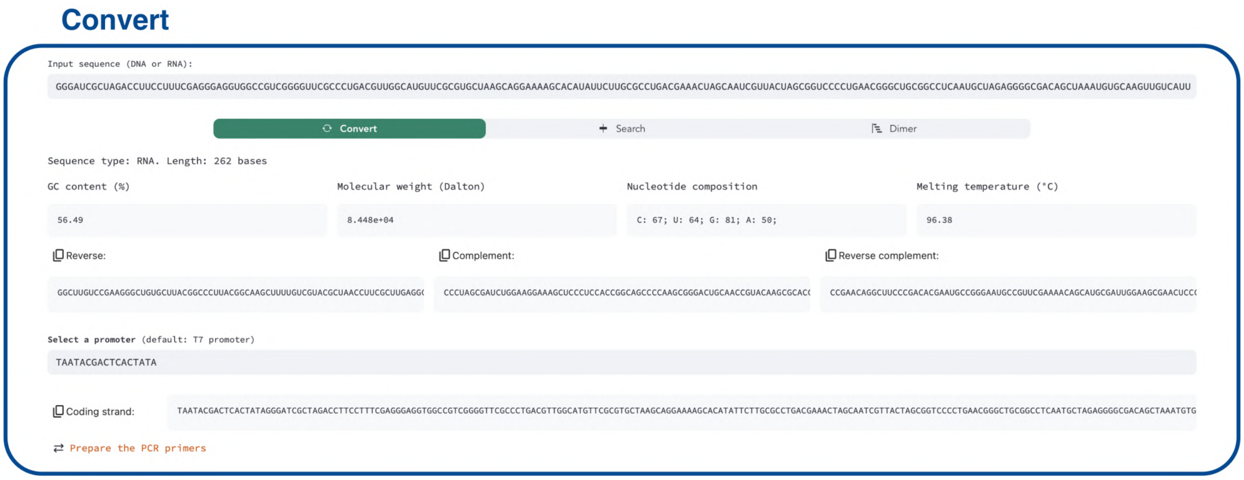

Figure S4: Screenshot of the pyFuRNAce 'Convert' page where RNA sequences are converted into DNA templates, including the addition of a transcription promoter sequence. The DNA templates can then be ordered as linear fragments or plasmids from a commercial source or synthesized in the lab.

1.5 Figure S5: Screenshot of the Prepare page

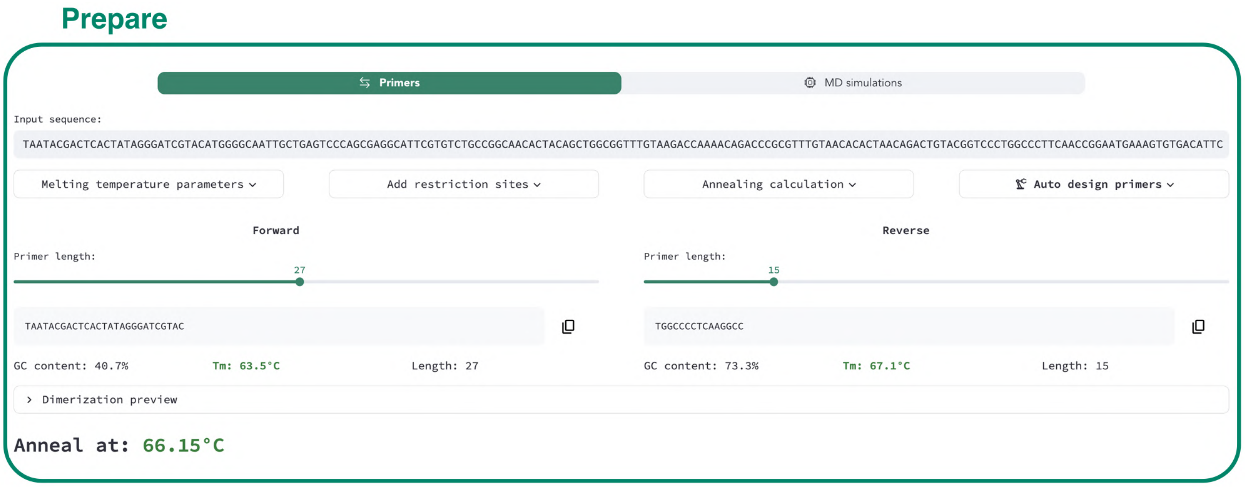

Figure S5: Screenshot of the pyFuRNAce 'Prepare' page supporting primer design and oxRNA coarse grained molecular dynamics simulations [3].

1.6 Figure S6: Agarose gel electrophoresis and plate reader measurements

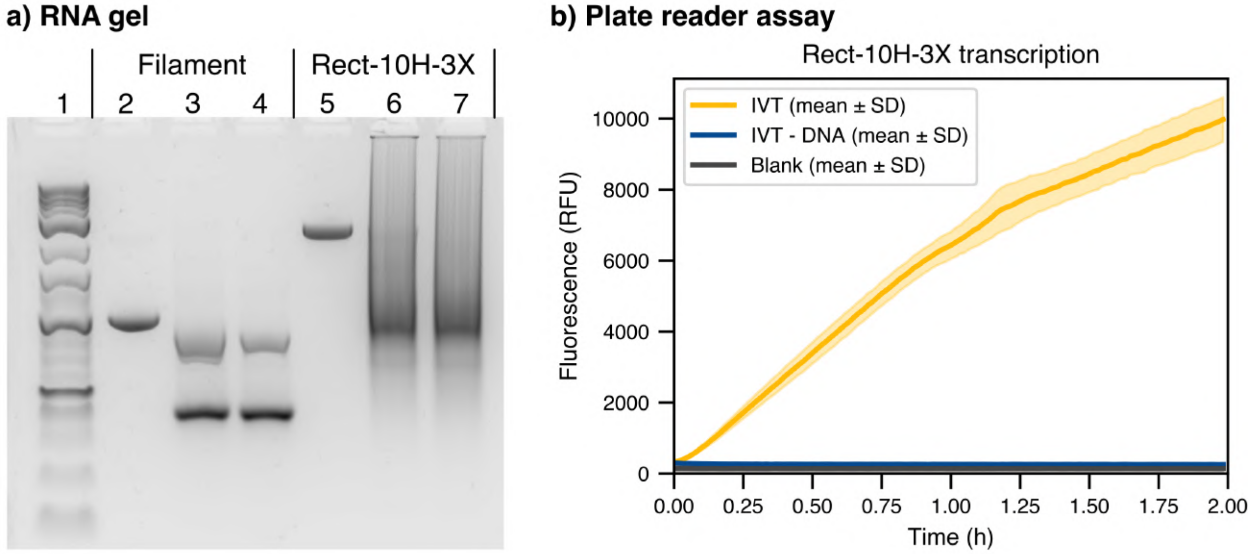

Figure S6: **a)** Agarose gel electrophoresis (1.75%, 1X TAE) of PCR amplified DNA (100 ng, Lanes 2 and 5) and 6 μL of in vitro transcription (IVT) mix, before (Lane 3 and 6) and after (Lane 4 and 7) DNase I-XT treatment to digest the DNA template (0.1 U/μL DNase I-XT, incubation for 30 min at 37°C). The two bands in the filament origami (Lanes 3 and 4) are likely the monomer (lower band) and the dimer (higher band). The band of the rectangular origami Rect-10H-3X (Lanes 6 and 7) is smeared probably due to the presence of DMSO from the DFHBI-1T dye. A 1 kb+ TriDye™ DNA ladder (NEB) was added in Lane 1. For each structure, IVT samples with or without DNase treatment were aliquoted from a fresh IVT reaction (n=1). The raw agarose gel image is provided in the Source Data file. **b)** Plate reader assay of Broccoli/DFHBI-1T fluorescence during transcription of the large rectangular RNA origami Rect-10H-3X (see Materials and Methods). Data is plotted as mean with standard deviation. The plate reader assay was run in triplicates (n=3), and the raw measurements are provided in the Source Data file.

1.7 Figure S7: AFM of filament-forming RNA origami

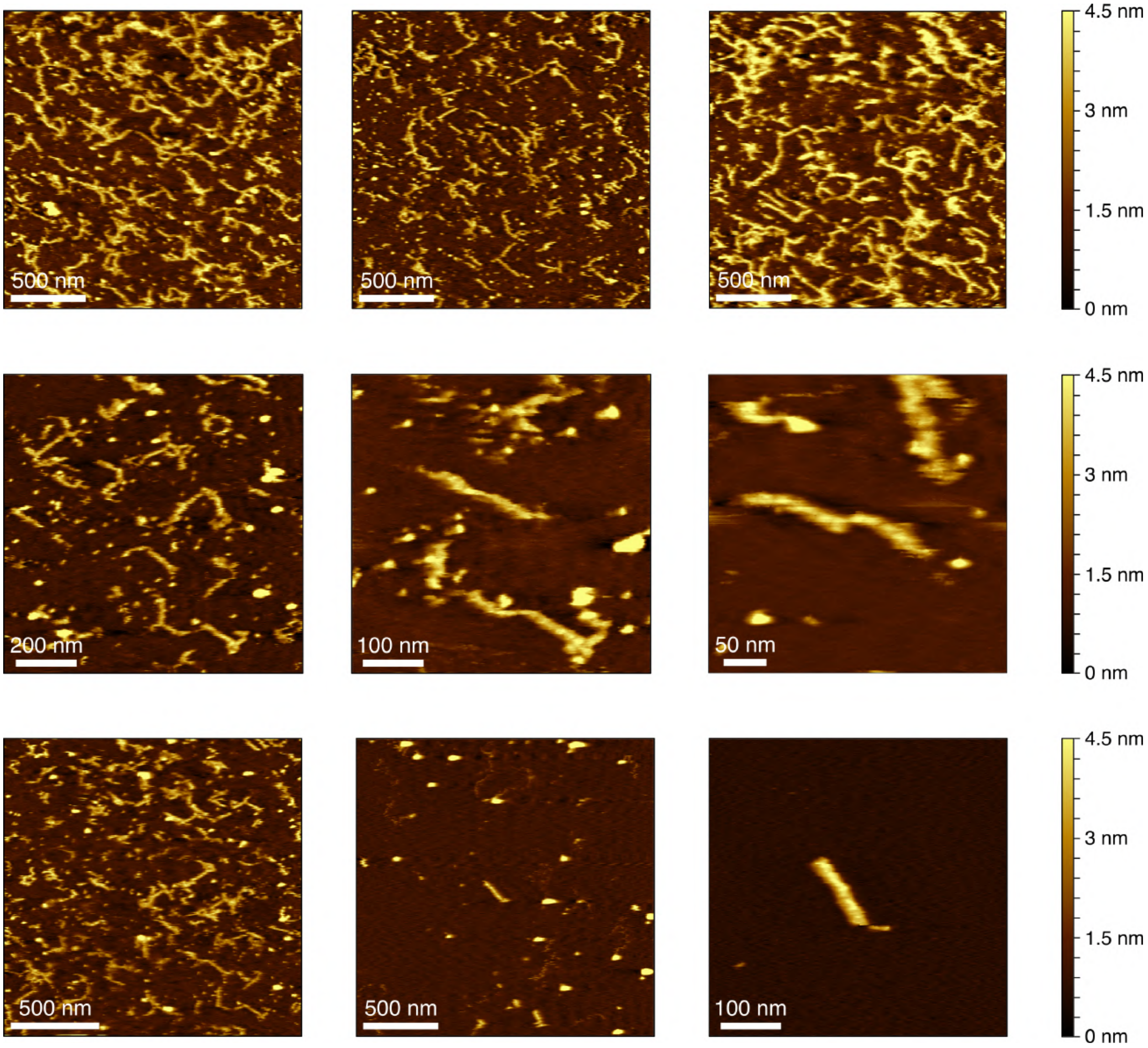

Figure S7: Additional AFM images of the filament-forming RNA origami design.

1.8 Figure S8: AFM of large rectangular RNA origami

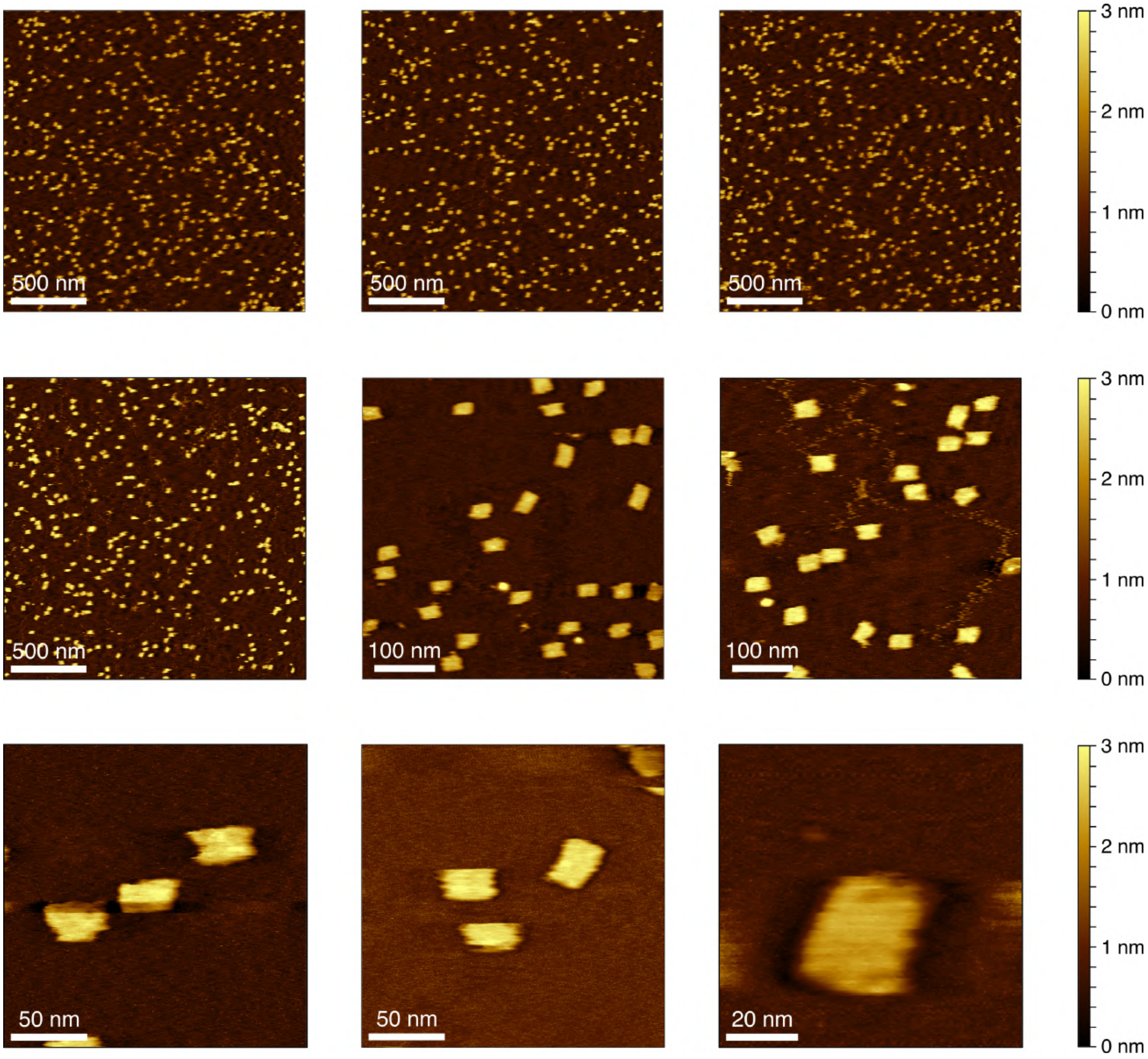

Figure S8: Additional AFM images of the large RNA origami rectangle Rect-10H-3X.

1.9 Figure S9: Yield estimation

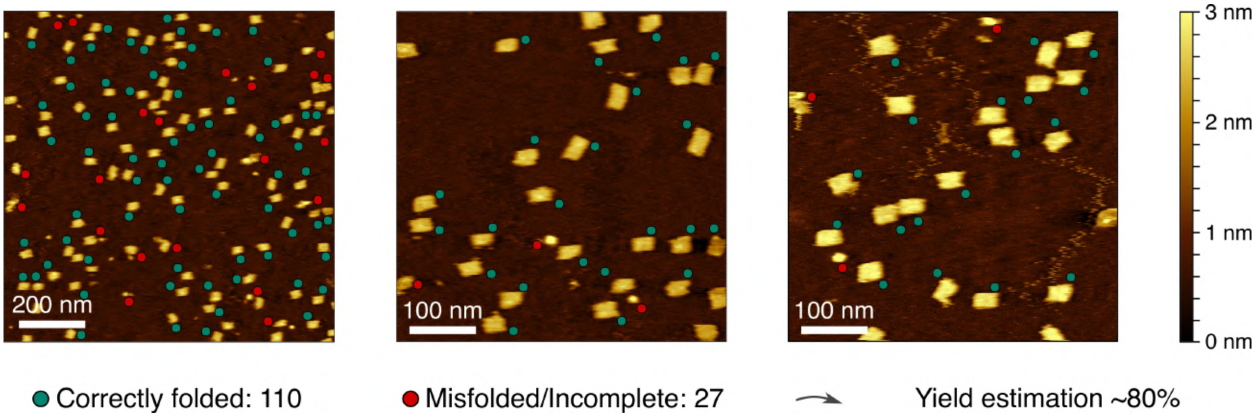

Figure S9: Annotated AFM images used to classify individual RNA origami structures as either correctly folded (green dots) or misfolded/incomplete (red dots). The annotations were manually added to quantify folding yields.

1.10 Figure S10: RNA droplet variants

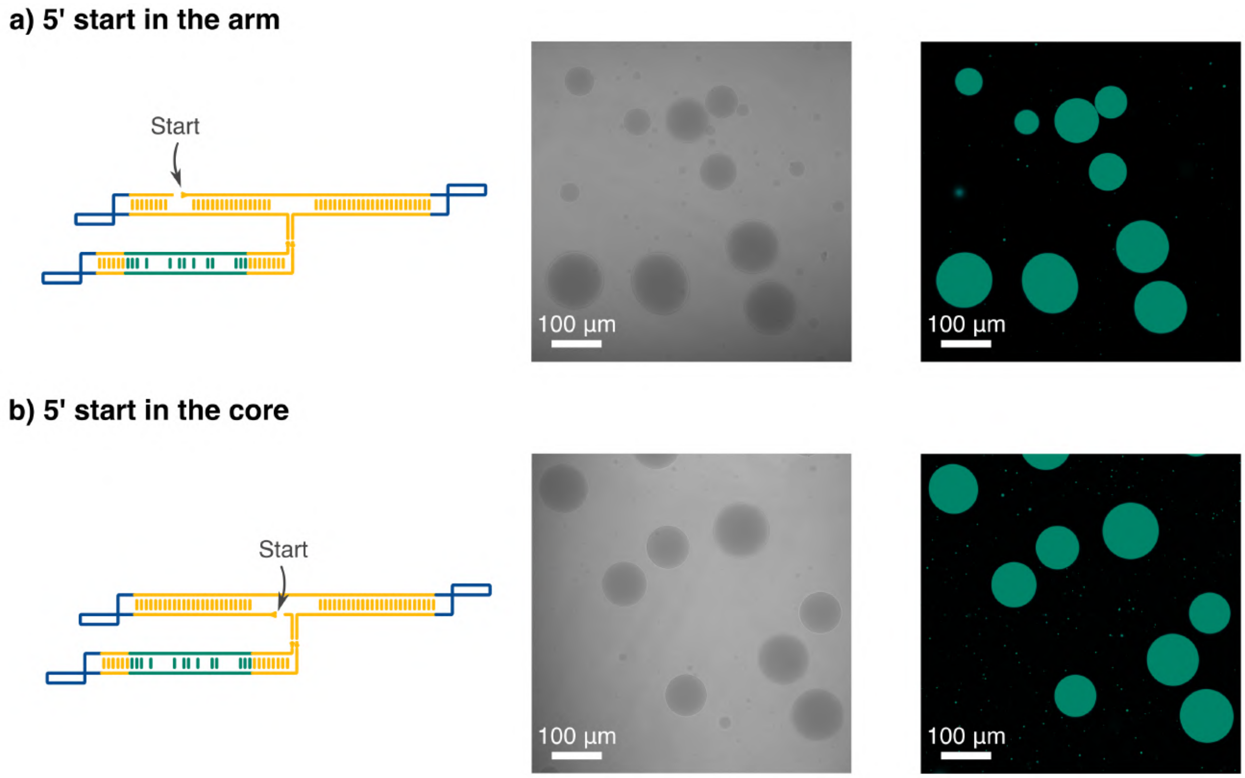

Figure S10: Comparison of droplets formation inserting the 5' start in the arm (**a**) or in the core (**b**) of the nanostar. The start position (and hence the position of the nick) does not seem to influence droplet formation significantly. The yellow color of the blueprint indicates a double helix, the blue color indicates a kissing loop, and the green color indicates the Broccoli aptamer.

2 Supplementary Videos

2.1 Video S1: Tutorial video

This video demonstrates the functionality of the pyFuRNAce web app interface, offering guidance to novice users. It starts by constructing a simple RNA origami by sequentially adding motifs predefined by pyFuRNAce (Tetraloops, Stems and Dovetails), changing their properties in the motifs menu and visualizing the blueprint and 3D origami structure. Then the 'Make a simple origami' popover is used to design a three-helices origami, and a pepper aptamer is inserted into the structure and visualized in 3D. The workflow continues to sequence generation, where the Revolvr algorithm produces a suitable RNA sequence. Once the RNA sequence is ready, the 'Convert' page produces the DNA template, adding a promoter for the T7 RNA polymerase. In the end, the 'Prepare' page is used for primer design, with the option to select the annealing temperature for the PCR.

3 Supplementary Notes

3.1 Note S1: Comparison of RNA design tools

Table 1: Feature comparison of pyFuRNAce with existing RNA design tools

	**pyFuRNAce**	**ROAD**	**Tiamat**	**Assemble2**	**Nanotiler**	**DNAForge**	**pyDAEDALUS**
Target structure	Co-transcriptional RNA origami	Co-transcriptional RNA origami	PX-based DNA/RNA origami	RNA nanostructures	RNA architectonics	Various wireframe	Scaffolded wireframe
Sequence optimization algorithm	Revolvr	Revolvr	MC algorithm	✗	✗	✗	✗
Folding included	✓ (ViennaRNA)	✓ (ViennaRNA)	✗	✗	✗	✗ (NUPACK)	✗ (NUPACK)
Non-helical motifs	✓	✓	✗	✓	✓	✗	✗
GUI	✓	✗	✓	✓	✓	✓	✗ (Athena)
Scripting API	✓	✗	✗	✗	✗	✗	✓
OS supported	All	Unix-like	Windows	All	All	All	All
Last update (as of 08.05.25)	2025	2024	~ 2019	2018	2012	2025	2024
Open source	✓	✓	✗	✓	✗	✓	✓
Language	Python	Perl	C++	Java	Java	TypeScript	Python

Over the years there have been a variety of software tools for RNA nanostructure design, each of which was developed to fulfill a target design niche. Some early tools, which have not been updated recently, include Tiamat, Assemble2 and Nanotiler. Tiamat is a Windows-only, free-form design tool which makes it easy to lay out helices in 3D space. It has primarily been used for DNA nanostructure design, and has been especially successful in designing DNA origami gridiron[4] and wireframe structures[5], which would not be possible in lattice-based tools such as CaDNAno[6]. Since Tiamat only supports helical sections, it was relatively easy to modify the representation to match the periodicity and spacing of A-form helices instead of B-form, allowing the tool to be used to design large PX RNA origami structures [7]. Its main weakness is that it lacks support for more complex motifs than helices – a key benefit of folding RNA – while also being more difficult to design large, complex structures in than lattice-based tools.

Assemble2[8, 9] and Nanotiler[10] are fragment assembly tools which have been used by the RNA architectonics community to build 3D structures from fragments mined from the PDB via homology searches. Nanotiler mostly been used to assemble small, compact RNA structures; for example Afonin *et. al* used Nanotiler to design 10-stranded RNA cubes[11]. Note that such structures require annealing, unlike co-transcriptional RNA origami. Assemble2 was a popular tool for model building in the RNA structural biology field (though its use has fallen off in recent years). However, it has also seen use in RNA nanotechnology; for example, Geary *et. al* used it to create the models in the original co-transcriptional RNA origami paper [12]. However, none of these tools have been updated in the last five years, and sometimes require some software wrangling to install on modern systems.

DNAForge[13] and pyDAEDALUS[14] are tools for converting arbitrary wireframe designs into DNA and RNA wireframe structures. DNAForge implements many previously published DNA and RNA routing algorithms into an oxView-like[15] web interface. It can rapidly convert wireframe meshes into DNA or RNA via the chosen algorithm. For DNA it can generate either scaffolded or scaffold-free routings, while for RNA all options are single-stranded and held together by internal kissing loops, much like the target use case of pyFuRNAce. The SteRNA routing method included in DNAForge was previously used to design RNA polyhedra[16], though the full GUI tool has not been out long enough to be used in other publications yet. The DAEDALUS routing algorithm generates scaffolded wireframe structures with two helices-per-edge. This offers improved stiffness over the single-helix-per-edge designs generated by the DNAForge algorithms at the cost of requiring more monomers per structure. It is offered as part of the ATHENA[17] package for DNA wireframe design. A modified version of DAEDALUS was used to make DNA-RNA hybrid structures, where many RNA sequences, including an mRNA and a 23S rRNA were folded using DNA staples[18]. One challenge for both of these tools is that they do not include sequence optimization algorithms. DNAForge offers input files to use NUPACK[19] to generate sequences, while DAEDALUS anticipates the use of a scaffold which enforces sequence.

ROAD can be considered the conceptual foundation for pyFuRNAce. We use the same design paradigm, fragment assembly on a lattice, and the same sequence-assignment, the multi-stage optimiser Revolvr. The major optimisation of pyFuRNAce over ROAD is the integration of all the different aspects of design, which in ROAD were partially handled by individual Perl scripts, into a single package with a graphical user interface. It offers a streamlined manipulation of the design data, which had to be done manually via text files in ROAD. PyFuRNAce also provides multiple features, including optimised origami design function, motif definition, nick-point optimisation, and primer design, which had to be handled by hand or other tools when using ROAD. This enables pyFuRNAce to successfully realize larger RNA origami assemblies as demonstrated here and to lower the entrance barrier to RNA origami for novice users.

3.2 Note S2: Automated primer design

The automated primer design in pyFuRNAce uses an iterative algorithm to identify forward and reverse primers with optimized properties for PCR amplification. The user specifies a target melting temperature, and primers are generated from the 5' ends of the template sequence and its reverse complement.

The algorithm starts with a minimal primer length (10 nucleotides) and iteratively extends the primer until the calculated melting temperature (Tm) falls within an acceptable range (within ±2.5°C of the target Tm). For each candidate primer, a scoring function evaluates:

- **3'-end stability:** +1 point each if the last two nucleotides are G or C.
- **Primer length:** +1 point if between 18–30 nucleotides; penalty if shorter than 18 nucleotides.
- **GC content:** +1 point if GC content is between 40–60%.
- **Melting temperature accuracy:** Penalty proportional to the deviation from the target Tm.
- **Self-dimerization:** Penalty based on the number of base pairs in the self-dimer, normalized by primer length.

Once all candidates are evaluated, the forward and reverse primers with the highest scores are selected and displayed. If no suitable primer is identified, an error message is shown, recommending manual primer selection.

It should be noted that the calculated melting temperature and the optimal annealing temperature are sensitive to the concentrations used, buffer conditions, and the underlying thermodynamic model. These parameters can be adjusted within the pyFuRNAce user interface to better reflect specific experimental conditions. However, if buffer compositions are unknown or kit-specific, it is recommended to cross-validate the melting temperatures using external calculators provided by the respective PCR kit manufacturers.

3.3 Note S3: Software limitations and workarounds

Table 2: Software dependencies used in pyFuRNAce, including required versions for stable support, usage context, licensing, and type of integration. Integration types are: `import` for dependencies installed with pip and used via standard Python imports, and `embedded` for libraries whose source code is included directly in the project repository.

Dependency	Version	Used In	License	Integration
pytest	8.3.3	testing	MIT	import
numpy	1.26.4	Design.Core.Coords	BSD	import
scipy	1.15.2	Design.Core.Coords	BSD	import
oxDNA-analysis-tools (OAT)	2.1	Design.Strand/Motif/Origami	GPL 3	import
ViennaRNA	2.6.4	Generate	Custom free license	import
Revolvr (from ROAD)	/	Generate	MIT	embedded
biopython	1.85	Prepare.Utils	BSD 3	import
streamlit	1.44.1	App	Apache 2.0	import
matplotlib	3.10.1	App.Pages.Design	PSFL	import
st-click-detector	0.1.3	App.Pages.Design	MIT	import
streamlit-code-editor	0.1.22	App.Pages.Design	MIT	import
streamlit-option-menu	0.4.0	App.Pages.Design	MIT	import
st_oxview	0.1.3	App.Pages.Design	GPL 3	import
colour	0.1.5	App.Pages.Generate	BSD	import

PyFuRNAce folding barrier estimation

To support cotranscriptional folding, the design of RNA origami should carefully avoid kinetic traps and steric hindrance. In the ROAD paper [20], a folding barrier penalty was introduced to account for a specific type of cotranscriptional steric hindrance. In particular, the RNA origami structures rely on duplexes with adjacent helices connected through 180° kissing loops (which form pseudoknots). For successful cotranscriptional folding, the transcription complex (DNA template, T7 RNA polymerase, and nascent RNA strand) must have sufficient spatial freedom for the RNA to assemble into duplexes. If the neighboring kissing loops are fully paired too early, then one of the strands which makes up the duplex is constrained at both ends, leading to steric hindrance which may prevent proper helix formation. This type of folding barrier has been further studied by Orponen et al. [21].

The folding barrier calculation in ROAD and pyFuRNAce highlights such cases: if an RNA duplex is expected to form only after adjacent kissing loops have already paired, the folding process may be hindered. To approximate this, the algorithm assumes that kissing loops do not pair instantaneously, introducing a default delay of 150 nucleotides during which no barrier is applied. Beyond this, two types of barriers are identified based on the length of the helix:

- **Weak barrier**: ≤ 5 bases (approximately half a helix turn), penalty +1.
- **Strong barrier**: > 5 bases, penalty +2.

To reduce folding barriers, three strategies are useful:

- **Use short dovetails rather than long dovetails,** since short dovetails require less than half a helix turn and thus lead to weaker barriers.
- **Change the 5′ start position of the structure,** which alters strand routing and can reduce the number of kissing loops between complimentary sequences. This optimization is available in both the pyFuRNAce GUI and API.
- **Modify the placement of continuous stems and kissing loops in the RNA origami structure,** which also changes routing. Since this requires altering the origami structure itself, it is not

implemented as a direct function, but we provide a Python script in the documentation to assist with this.

Ultimately, cotranscriptional folding depends on many factors beyond this simplified model. While the folding barrier calculation is a useful heuristic for estimating cotranscriptional feasibility, our experimental validation did not reveal a clear correlation between predicted penalties and folding outcomes. At present, the final judgment still rests with the designer and further experimental characterization is needed to fully understand the kinetic landscape of RNA origami folding.

Revolvr

For sequence generation and optimization, we use the Revolvr program from ROAD [20]. Briefly, Revolvr implements a series of optimization steps, first assigning a random sequence which has the complementarity of the target dot-bracket string, followed by mutation steps interleaved with calls to ViennaRNA's RNAfold, until the predicted secondary structure matches the target structure. It then continues to mutate to reduce sequence symmetry (minimizing non-target complementarity and repeated subsequences) and to remove common restriction enzyme sites. Finally, kissing loop (KL) sequences are orthogonalized to prevent unintended multimerization and loop closure. Revolvr introduces a number of limitations which are inherited by pyFuRNAce:

- **Optimization failure of structures containing repeated fixed sequences.** If there are more than four copies of a fixed sequence in a structure, Revolvr optimization will often get stuck during sequence-symmetry minimization. This is because the fixed sequence plus one base will fail the test for repeated sequences.

  Solution: By extending the fixed sequences by unequal amounts of random sequence, repeats of the exact same sequence in the optimization region can be avoided.

- **No multimeric structures.** Revolvr can only perform optimization of one strand at a time.

  Solution: Optimization of multi-strand assemblies needs to be done in multiple steps. The user can also replace external KLs with continuous duplexes and optimize the combined structure, replacing the continuous duplex with separate KLs after optimization.

- **Timeout during optimization of large structures.** Revolvr can be very slow at optimiztion large structures, and due to server constraints the pyFuRNAce webserver has a timeout of 2 hours.

  Solution: When running the optimisation through Python scripts, the Revolvr timeout can be set to an arbitrary value. For the 2.5kb structure shown in Figure 5, we used a 24-hour timeout (See Supplementary Note S5). Designs with particular constraints (e.g. short stems, such as dovetails or long unpaired regions) are typically hard to design, and Revolvr might fail repeatedly. PyFuRNAce also provides a function to run multiple instances of Revolvr in parallel, called "parallel_road", which can speed up the optimisation, but requires multiple compute cores. Optimisation through Python scripts can also be run on a high-performance computing cluster, even though the parallelism is shared exclusively on one node.

- **Lack of fine control over KL energies.** As RNA structures grow in size and complexity, the number of KL needed to fold the complete structure naturally also increases. By specifying the interaction energy of each KL pair, RNA designers could gain some control over the folding pathway and assembly order of multimeric structures, enabling larger, more complex structures.

  Solution: Users can identify orthogonal KLs with the target energies ahead of time and manually insert the desired sequences. The KL sequence can be inserted when the motif is added or edited later in the "Edit" tab. We frequently use Table S4 from Geary *et. al* 2021 [20], which lists KL energies, for this purpose.

We are investigating novel sequence optimization strategies which overcome these limitations. PyFuRNAce is written with this eventuality in mind, and the sequence optimization algorithm is something which can be integrated later with a list of available algorithms.

ViennaRNA

RNAfold is a dependency of Revolvr, used to predict the secondary structure of a given RNA sequence. It is part of the popular ViennaRNA[1] suite of bioinformatic tools. The folding algorithm is a dynamic programming algorithm[22], which uses the Turner nearest neighbor model[23] to predict the minimum free energy fold of the sequence. There are two major limitations introduced by RNAfold to the optimization of RNA structures:

- **Lack of pseudoknot prediction.** Unlike computing nested pairs which can be computed in polynomial time, pseudoknot enumeration is an NP-complete[24] problem. Because of this, RNAfold, like most other secondary structure prediction tools does not consider pseudoknots. Internal KLs, however, are pseudoknots, which is why Revolvr has to perform KL orthogonalization in a separate step. Unless $P = NP$, this will remain a problem and we can only hope to develop better heuristic algorithms for KL sequence assignment for all but the smallest structures.

- **Lack of stabilizing tertiary interactions.** All RNA sequence optimization discussed here is performed at the secondary structure level—only Watson-Crick-Franklin base pairing is considered. This is the most common type of base-pairing in RNA structures; However, RNA can form a huge variety of additional stabilizing hydrogen bonds and pi-pi stacking interactions, meaning that this approximation is generally an underestimate of the free energy of any given RNA structure. There are two common motifs where this can pose problems in RNA origami design. First, the 180° KLs have an interaction in which two adenines stack across the major groove of the 6-base helix, stabilizing the structure. Second, many flourescent aptamers, in particular, contain G-quadruplexes where four guanines interact via Hoogsteen-edge interactions. This means that predicted structures from RNAfold involving aptamers are generally inaccurate. We have not seen this cause major problems with designed structures, but it's important to keep in mind as a potential confounding factor. This is not something that we have an immediate solution for, but if co-transcriptional RNA origami becomes a widely-adopted technology, quantifying the true energies of 180° KLs would be beneficial.

oxDNA Analysis Tools

PyFuRNAcs uses oxDNA Analysis Tools (OAT) to convert 3D models from the xoDNA representation (natively supported) to PDB.

- **Inaccurate sub-nucleotide features** As an anisotropic one-bead-per-nucleotide model, oxDNA's file format only tracks the center of mass and orientation of each base, but lower level atomic information is lost. When users download PDB files from pyFuRNAce, the files are generated from the oxDNA representation via OAT's converter, which assembles ideal all-atom representations of nucleotides to the reference frame of the coarse-grained representation. These all-atom representations come from an NMR structure of an RNA helix (PDB ID: 2jxq) [25]. This is not an optimized structure and should be considered a low-resolution model.

  Solution: Before additional model building or all-atom molecular dynamics simulations, we usually refine the all-atom PDB structures with QRNAS [26] using default settings.

3.4 Note S4: OxRNA Molecular Dynamics Simulation Setup

The molecular dynamics simulations are based on the default input files provided by the oxDNA developers, available at: `https://github.com/lorenzo-rovigatti/oxDNA/tree/master/analysis/example_input_files`.

To account for the specific structure of co-transcriptional RNA origami, which relies on the stability of short pseudoknots, an additional intermediate step was introduced between the relaxation and production phases.

The oxRNA simulation protocol integrated into pyFuRNAce consists of the following steps:

- **Monte Carlo relaxation**, with active external forces enforcing base pairing, to guide the structure into a folded conformation.

- **Molecular dynamics relaxation**, still maintaining base-pairing forces.
- **Molecular dynamics equilibration**, with base-pairing forces applied only to pseudoknotted regions.
- **Molecular dynamics production run**, without external forces and with sequence-dependent parameters enabled.

The simulation-ready topology, configuration, force field, and input files are automatically generated and provided as a compressed archive for direct use in oxRNA simulations.

3.5 Note S5: RNA nanostructure scripts

RNA filament

```
import pyfurnace as pf
origami = pf.Origami()
origami.append([]) # Add empty line

origami = pf.simple_origami(dt_list=[180, 90, 270, 270, 90, 180],
                            kl_columns=1,
                            main_stem=33,
                            add_terminal_helix=True,
                            align="left",
                            use_angles=True) # Create a simple origami

origami = origami.improve_folding_pathway(kl_delay=150)

motif = origami[(0, 6)] # motif slice: line 0, index 6
motif.length = 12

motif = origami[(0, 1)] # motif slice: line 0, index 1
motif.length = 13

motif = origami[(7, 1)] # motif slice: line 7, index 1
motif.length = 12

motif = origami[(7, 9)] # motif slice: line 7, index 9
motif.length = 13

origami.pop((0, 7)) # Delete motif

motif = pf.KissingLoop180(open_left = True, pk_index = "1")
origami.insert((0, 7), motif) # Add motif

origami.pop((0, 0)) # Delete motif

motif = pf.KissingLoop180(open_left = False, pk_index = "1'")
origami.insert((0, 0), motif) # Add motif

origami.pop((7, 10)) # Delete motif

motif = pf.KissingLoop180(open_left = True, pk_index = "2")
origami.insert((7, 10), motif) # Add motif

origami.pop((7, 0)) # Delete motif

motif = pf.KissingLoop180(open_left = False, pk_index = "2'")
origami.insert((7, 0), motif) # Add motif
```

Large RNA origami Rect-10H-3X

```
import pyfurnace as pf
origami = pf.Origami()
origami.append([]) # Add empty line
origami = pf.simple_origami(dt_list=[180] * 8,
                            kl_columns=3,
                            main_stem=33,
```

```
                               add_terminal_helix=True,
                               align="left",
                               use_angles=True) # Create a simple origami

origami = origami.improve_folding_pathway(kl_delay=150)

motif = pf.Broccoli().flip(1, 1)
origami.insert((0, 11), motif) # Add motif

motif = pf.Stem(length = 5, wobble_interval = 7, wobble_tolerance = 3,
                wobble_insert = 'middle', strong_bases = True)
origami.insert((0, 12), motif) # Add motif

###
### CODE FOR SEQUENCE OPTIMIZATION:
###
from pyfurnace.generate import generate_road

ORIGAMI_NAME = 'Rect_10H_3X' # name for optimization and formats
TIMEOUT = 60 * 60 * 24 # ROAD timeout in seconds, e.g. (60*60*24) is 24 hours

seq_constraints = str('GGGA' + origami.sequence[4:])

opti_sequence = generate_road(origami.structure,
                              seq_constraints,
                              origami.pseudoknots,
                              name=ORIGAMI_NAME,
                              directory='Rect_10H_3X',
                              timeout=TIMEOUT,
                              verbose=True,
                              )

print('Optimized␣sequence:', opti_sequence)

origami.sequence = opti_sequence
origami.save_text(ORIGAMI_NAME)
origami.save_3d_model(ORIGAMI_NAME, pdb=True)
```

RNA Droplet (5-in-arm)

```
import pyfurnace as pf

origami = pf.Origami(align='first')
palindr_kl = pf.KissingLoop(sequence='AUCGCGAAA')
origami.append([palindr_kl.copy(),
                pf.Stem(sequence='GNNNKNNC'),
                pf.start_end_stem(),
                pf.Stem(sequence='GGNNNKNNNNNNNNNKC'),
                pf.Motif.from_structure('.&', 'U&').flip(),
                pf.Dovetail(0, up_cross=False),
                pf.Motif.from_structure('.&.', 'U&U'),
                pf.Stem(sequence='GNNKNNNNNKNNNNNKNNNNNKNNC'),
                palindr_kl.copy().flip()
                ])
origami.append([palindr_kl.copy(),
                pf.Stem(sequence='GNNKN'),
                pf.Broccoli(),
                pf.Stem(sequence='NNNKNNNG'),
                pf.stem_cap_link().flip()
                ])
```

RNA droplet (5-in-core)

```
import pyfurnace as pf

origami = pf.Origami(align='first')
palindr_kl = pf.KissingLoop(sequence='AUCGCGAAA')
origami.append([palindr_kl.copy(),
```

```
                pf.Stem(sequence='GNNNKNNC'),
                pf.Stem(sequence='GGNNNKNNNNNNNNNKC'),
                pf.start_end_stem().flip(),
                pf.Dovetail(0, up_cross=False),
                pf.Motif.from_structure('.&.', 'U&U'),
                pf.Stem(sequence='GNNKNNNNNKNNNNNKNNNNNKNNC'),
                palindr_kl.copy().flip()
                ])
origami.append([palindr_kl.copy(),
                pf.Stem(sequence='GNNKN'),
                pf.Broccoli(),
                pf.Stem(sequence='NNNKNNNG'),
                pf.stem_cap_link().flip()
                ])
```

Supplementary References

[1] Ronny Lorenz et al. "ViennaRNA Package 2.0". In: *Algorithms for molecular biology* 6 (2011), pp. 1–14.

[2] Ivo L Hofacker et al. "Fast folding and comparison of RNA secondary structures". In: *Monatshefte für chemie* 125 (1994), pp. 167–167.

[3] Petr Šulc et al. "A nucleotide-level coarse-grained model of RNA". In: *The Journal of Chemical Physics* 140.23 (2014).

[4] Dongran Han et al. "DNA gridiron nanostructures based on four-arm junctions". In: *Science* 339.6126 (2013), pp. 1412–1415.

[5] Fei Zhang et al. "Complex wireframe DNA origami nanostructures with multi-arm junction vertices". In: *Nature nanotechnology* 10.9 (2015), pp. 779–784.

[6] Shawn M Douglas et al. "Rapid prototyping of 3D DNA-origami shapes with caDNAno". In: *Nucleic Acids Research* 37.15 (2009), pp. 5001–5006.

[7] Dongran Han et al. "Single-stranded DNA and RNA origami". In: *Science* 358.6369 (2017), eaao2648.

[8] Fabrice Jossinet, Thomas E Ludwig, and Eric Westhof. "Assemble: an interactive graphical tool to analyze and build RNA architectures at the 2D and 3D levels". In: *Bioinformatics* 26.16 (2010), pp. 2057–2059.

[9] Fabrice Jossinet. "Assemble2: an interactive graphical environment dedicated to the study and construction of RNA architectures". In: *2015 IEEE 1st International Workshop on Virtual and Augmented Reality for Molecular Science (VARMS@ IEEEVR)*. IEEE. 2015, pp. 37–38.

[10] Eckart Bindewald et al. "Computational strategies for the automated design of RNA nanoscale structures from building blocks using NanoTiler". In: *Journal of Molecular Graphics and Modelling* 27.3 (2008), pp. 299–308.

[11] Kirill A Afonin et al. "In vitro assembly of cubic RNA-based scaffolds designed in silico". In: *Nature nanotechnology* 5.9 (2010), pp. 676–682.

[12] Cody Geary, Paul WK Rothemund, and Ebbe S Andersen. "A single-stranded architecture for cotranscriptional folding of RNA nanostructures". In: *Science* 345.6198 (2014), pp. 799–804.

[13] Antti Elonen et al. "DNAforge: a design tool for nucleic acid wireframe nanostructures". In: *Nucleic Acids Research* 52.W1 (2024), W13–W18.

[14] Rémi Veneziano et al. "Designer nanoscale DNA assemblies programmed from the top down". In: *Science* 352.6293 (2016), pp. 1534–1534.

[15] Erik Poppleton et al. "Design, optimization and analysis of large DNA and RNA nanostructures through interactive visualization, editing and molecular simulation". In: *Nucleic Acids Research* 48.12 (2020), e72–e72.

[16] Antti Elonen et al. “Algorithmic design of 3D wireframe RNA polyhedra”. In: *ACS nano* 16.10 (2022), pp. 16608–16616.

[17] Hyungmin Jun et al. “Rapid prototyping of arbitrary 2D and 3D wireframe DNA origami”. In: *Nucleic Acids Research* 49.18 (2021), pp. 10265–10274.

[18] Molly F Parsons et al. “3D RNA-scaffolded wireframe origami”. In: *Nature Communications* 14.1 (2023), p. 382.

[19] Mark E Fornace et al. “NUPACK: Analysis and design of nucleic acid structures, devices, and systems”. In: *ChemRxiv* (2022).

[20] Cody Geary et al. “RNA origami design tools enable cotranscriptional folding of kilobase-sized nanoscaffolds”. In: *Nature chemistry* 13.6 (2021), pp. 549–558.

[21] Pekka Orponen, Shinnosuke Seki, and Antti Elonen. “Secondary Structure Design for Cotranscriptional 3D RNA Origami Wireframes”. In: *International Conference on DNA Computing and Molecular Programming*. Schloss Dagstuhl-Leibniz-Zentrum für Informatik. 2025, pp. 1–18.

[22] Michael Zuker and David Sankoff. “RNA secondary structures and their prediction”. In: *Bulletin of mathematical biology* 46.4 (1984), pp. 591–621.

[23] Tianbing Xia et al. “Thermodynamic parameters for an expanded nearest-neighbor model for formation of RNA duplexes with Watson- Crick base pairs”. In: *Biochemistry* 37.42 (1998), pp. 14719–14735.

[24] Rune B Lyngsø and Christian NS Pedersen. “Pseudoknots in RNA secondary structures”. In: *Proceedings of the fourth annual international conference on Computational molecular biology*. 2000, pp. 201–209.

[25] Lukasz Popenda, Ryszard W Adamiak, and Zofia Gdaniec. “Bulged adenosine influence on the RNA duplex conformation in solution”. In: *Biochemistry* 47.18 (2008), pp. 5059–5067.

[26] Juliusz Stasiewicz et al. “QRNAS: software tool for refinement of nucleic acid structures”. In: *BMC structural biology* 19.1 (2019), p. 5.
