# Supplementary material for: PyFuRNAce: an integrated design engine for RNA origami
Source: Nat Commun. 2025 Dec 1;16:10815. doi: 10.1038/s41467-025-66290-x (PMC12669765; doi:10.1038/s41467-025-66290-x)
Supplement: Supplementary file 2 — Description of Additional Supplementary Files [file 41467_2025_66290_MOESM2_ESM.pdf]

**Supplementary Video 1**

Demonstrates the functionality of the *pyFuRNAce* web app interface, offering guidance to novice users. The video begins with the construction of a simple RNA origami by sequentially adding motifs predefined by *pyFuRNAce* (Tetraloops, Stems, and Dovetails), modifying their properties in the motifs menu, and visualizing both the blueprint and 3D origami structure. It then shows the use of the 'Make a simple origami' popover to design a three-helix origami, followed by insertion of a pepper aptamer and 3D visualization. The workflow proceeds to sequence generation using the Revolvr algorithm, producing a suitable RNA sequence. Finally, the 'Convert' page is used to generate the DNA template with a T7 promoter, and the 'Prepare' page illustrates primer design with user-defined annealing temperatures for PCR.

**Supplementary Data 1**

Contains the DNA sequences of the templates and primers used for cotranscriptional folding of the RNA nanostructures.
